# Reviewed article: Lourenço ARP, Coutinho TAA, Monteiro LS, Botelho LV, Pontes PV, Sperandio N. Nutritional status of preschoolers in the public school system of Macaé, Rio de Janeiro: a panel study conducted between 2012-2014 and 2017-2019 in the context of the oil crisis and the implementation of educational actions based on the Dietary Guidelines for the Brazilian Population. Epidemiol Serv Saude. 2025:34;e20240386

**DOI:** 10.1590/S2237-96222025v34e20240386.a

**Published:** 2025-08-04

**Authors:** Larissa Loures Mendes

**Table:** 

Rating	Excellent	Good	Average	Below average	Poor
Interest				✓	
Quality				✓	
Originality				✓	
Overall				✓	

**Table:** 

Questionnaire	Yes	No	Not applicable
Does the manuscript contain new and significant information to justify publication?		✓	
Does the Abstract (Summary) clearly and accurately describe the content of the article?		✓	
Is the problem significant and concisely stated?		✓	
Are the methods described comprehensively?	✓		
Are the interpretations and conclusions justified by the results?		✓	
Is adequate reference made to other work in the field?	✓		

## Manuscript Structure

Length of article is: Adequate

Number of tables is: Adequate

Number of figures is: Adequate

Please state any conflict(s) of interest that you have in relation to the review of this paper (state “none” if this is not applicable): None

## Comments

Prezados Autores,

Embora a temática do estudo seja relevante e atual, as limitações metodológicas apresentadas impedem a aceitação do artigo em sua forma atual. A realização das modificações sugeridas podem fortalecer o estudo e aumentar a confiabilidade dos resultados para uma futura submissão.

A proposta do estudo, que busca avaliar o impacto de um programa de educação nutricional em pré-escolares, é de grande relevância e alinha-se com as diretrizes atuais de promoção da saúde. No entanto, o delineamento metodológico apresentado apresenta algumas limitações que impedem a obtenção de evidências robustas para responder à questão de pesquisa.

Principais Limitações:

Delineamento Transversal: A utilização de um delineamento transversal limita a capacidade de avaliar mudanças no estado nutricional ao longo do tempo. Para investigar o efeito de uma intervenção, como um programa de educação nutricional, é fundamental um delineamento longitudinal, que permita acompanhar os participantes em diferentes momentos e comparar os resultados antes e após a intervenção.

Amostra Não Probabilística: A utilização de uma amostra não probabilística restringe a generalização dos resultados para a população de pré-escolares da rede pública de Macaé. Uma amostra probabilística, como a aleatória simples ou estratificada, garante maior representatividade e permite inferências mais confiáveis sobre a população em estudo.

Análises Descritivas: As análises descritivas, embora importantes para caracterizar a amostra e os resultados da intervenção, não são suficientes para avaliar o impacto da educação nutricional no estado nutricional dos pré-escolares. É necessário o emprego de testes estatísticos adequados para comparar grupos e identificar diferenças significativas entre os momentos da avaliação.

Recomendações para Submissões Futuras:

Para que o estudo possa ser considerado para publicação em uma revista científica, os autores são incentivados a:

Revisar o delineamento metodológico: Adotar um delineamento longitudinal, com coleta de dados em pelo menos dois momentos (pré e pós-intervenção), para avaliar as mudanças no estado nutricional ao longo do tempo.

Ampliar a amostra: Utilizar uma amostra probabilística, com um tamanho de amostra adequado para garantir o poder estatístico necessário para detectar diferenças significativas.

Aprimorar as análises estatísticas: Empregar testes estatísticos apropriados para comparar grupos e identificar a associação entre as variáveis de interesse. Além das análises descritivas, realizar análises inferenciais, como testes t para amostras independentes ou pareadas, ou análise de variância (ANOVA), para avaliar o efeito da intervenção.

Controlar variáveis confundidoras: Considerar a influência de outras variáveis que podem afetar o estado nutricional dos pré-escolares, como nível socioeconômico, hábitos alimentares familiares e atividade física, e controlar esses fatores nas análises estatísticas.

